# Transfer of Problem Solving Skills from Touchscreen to 3D Model by 3- to 6-Year-Olds

**DOI:** 10.3389/fpsyg.2017.01586

**Published:** 2017-09-20

**Authors:** Joanne Tarasuik, Ana Demaria, Jordy Kaufman

**Affiliations:** ^1^School of Health Sciences, Swinburne University of Technology, Hawthorn VIC, Australia; ^2^Department of Early and Preschool Education, University of Rijeka Rijeka, Croatia

**Keywords:** children, multimedia, touchscreen, human–computer interaction, transfer of learning

## Abstract

Although much published research purports that young children struggle to solve problems from screen-based media and to transfer learning from a virtual to a physical modality, Huber et al. (2016)’s recent study on children solving the Tower of Hanoi (ToH) problem on a touchscreen app offers a clear counter example. Huber et al. (2016) reported that children transferred learning from media to the physical world. As this finding arguably differs from that of prior research in this area, the current study tests whether the Huber et al. (2016) results could be replicated. Additionally, we extended the scope of the Huber et al. (2016) work by testing a broader age range, including children as young as 3 years, and using a culturally distinct participant pool. The results of the current study verified Huber et al.’s (2016) conclusion that 4- to 6-year-old children are capable of transferring the ToH learning from touchscreen devices to the physical version of the puzzle. Children under 4 years of age, in contrast, showed little ability to improve at the ToH problem regardless of the practice modality—suggesting that a different problem-solving task is required to probe very young children’s ability to learn from touchscreen apps.

## Introduction

Touch screen devices such as tablet, computers, and smart phones provide adults and children with access to countless interactive apps, many of which claim to offer learning opportunities (Shuler, 2012). These claims, however, run counter to the literature underlying most media use guidelines published by government bodies and academic and medical organizations. Most published research suggests that while young children may learn a skill or problem-solving strategy from screen-based media, they struggle to apply this learning in a new non-screen-based context (Hirsh-Pasek et al., 2015).

For example, Zack et al. (2009, 2013) conducted button-pressing imitation experiments with 15-month-old children. In these experiments, an adult demonstrated a button-press with either a real physical button (3D modality or simply “3D”) or a virtual button presented on a touchscreen display (2D modality or simply “2D”). Examining whether children would imitate this action within and across modalities, Zack et al. (2009, 2013) reported that children were most likely to imitate an adult’s demonstrated action when the adult and child performed their actions in the same modality, i.e., children observe on a 2D screen and imitate on a 2D screen (2D-2D) or children observe on 3D object and imitate on the 3D object (3D-3D). In contrast, imitation was significantly impaired when the observation and imitation modalities differed (3D-2D and 2D-3D). Zack et al. (2009, 2013) concluded that although imitation skill can be learned in either modality, the imitation skill cannot effectively be transferred across modalities.

Similarly, using an imitation paradigm involving a puzzle assembly task, Moser et al. (2015) demonstrated a similar finding with 2.5- and 3-year-old children. Results revealed a transfer deficit (i.e., a drop in performance across modalities) when the children were required to imitate on the touch screen device what they observed on the felt board puzzle (3D-2D) and vice versa (2D-3D) compared to imitation that did not require a transfer across modalities. Interestingly, however, not all researchers find transfer deficits in their experiments. Chen and Siegler (2013) found that children as young as 2 years of age could learn to imitate a series of steps to solve a spatial problem using tools from a video presentation—therefore not showing the transfer deficit seen in the imitation studies described above. Chen and Siegler (2013) highlight that 2D content that conveys that there is a problem to be solved can be challenging for young children, and learning from video is increasingly difficult as the number of steps required to achieve a goal increases (Barr, 2010; Barnett, 2014). Furthermore, learning to solve problems is increasingly reliant upon engagement in the task as the task complexity increases (e.g., Bauer and Mandler, 1992).

In light of these potentially contradictory findings, Huber et al. (2016) (the reference study here), examined the extent to which a change in modality affected children’s learning of a problem-solving task. In that study, it was hypothesized that children would show significant transfer of learning from a 2D to 3D modality because solving the task requires engagement in the process, potentially overriding focus on superficial modality-based differences. Most initiation studies in contrast, require nothing during the “learning” phase apart from observation. Therefore, it is possible that children show a transfer deficit either because they are not sufficiently engaged in the learning process or that they find it easiest to imitate under conditions that are superficially similar to those seen during the demonstration.

Huber et al. (2016) examined 4- to 6-year-old children’s ability to transfer learning acquired while solving a Tower of Hanoi (ToH) problem on a touchscreen device to solving the standard physical version of the problem. The results were that, regardless of the modality in which a child practiced, children’s performance on the task improved significantly after practice. Indeed, there was no evidence that practicing on the physical version conferred any advantages over practicing with the 2D version as measured by final performance on a physical version test trial. These results suggest that children are able to transfer what they have learned from a touchscreen to “real-world” situation. Huber et al.’s (2016) finding stands out because transfer of problem solving skills from screen media 2D modality to the physical context of 3D modality is often claimed to be particularly difficult for young children (e.g., Schmidt and Vandewater, 2008). This raises the importance of replicating the Huber study to confirm its validity to our understanding of children’s learning from touchscreen media.

As such, the current investigation aimed to replicate the findings of Huber et al. (2016) (also referred to here as the “reference” study), hypothesizing comparable patterns of results for analyses including participants of the same age. Additionally, as the majority of the previous work has investigated children younger than the 4- to 6-years-olds studied in the reference study, the current study expanded the reference study’s age range to include younger children (from 3 years of age). Historically, studies of computer use have not examined children under 4 years because traditional desktop computer use requires cognitive and motor skills unavailable to younger children. However, with the rise of tablets, children are using computing devices as early as a child’s first year of life (Kabali et al., 2015; Tarasuik and Kaufman, 2017) which is reflected in the wide range of “educational” apps targeting parents of young children (Hirsh-Pasek et al., 2015). Our inclusion of this younger group aims to help fill this newly relevant gap.

Based findings of Chen and Siegler (2013), it was hypothesized that the younger children would also demonstrate transfer, provided that they could sufficiently improve at the problem-solving task over multiple trials in any modality. Also, the transfer of learning protocol used by Huber et al. (2016) in Australia was replicated in Croatia, using the same materials developed by Huber and colleague’s research team and the same physical materials and software (but with a different set of experimenters). Conditions replicated the reference study absent the condition where participants completed the task solely with the physical model, as the focus of the research was transferring learning across modalities.

## Materials and Methods

This experiment was designed to determine how experience with a problem-solving task in a particular modality (i.e., using physical “3D” vs. virtual “2D”) affects children’s improvement in performance in a new modality. The procedure for the current study largely replicates that used in Huber et al. (2016) in which children completed four trials on a three disk ToH puzzle. Specifically, the methods were designed to answer the questions: How does practice with a virtual puzzle transfer to performance with the traditional, physical puzzle?

### Participants

A total of 49 children (45% male) aged 3.1 to 6.5 years (*M* = 4.8, *SD* = 1.1) were included in the analysis. An additional six children participated but were excluded from analysis due to failure to follow instructions on any trial (*n* = 1) or failure to complete all l four trials in the experiment (*n* = 5). Croatian was the main language spoken by all children, although some attended English (*n* = 11) or Italian (*n* = 11) language classes, and none of the children were reported to have any additional health care needs. More than half of mothers and almost a third of father had completed a minimum of an undergraduate university qualification, and family income (in Croatian Kuna) was reported to be <kn50K with exception of one family whose reported income was kn75K < kn100K. The participants were recruited from a day care centre in Rijeka, a metropolitan city of Croatia.

### Materials

The experiment used the ToH problem solving puzzle, selected because of its extensive use with children as an assessment of problem solving, planning ability and executive functioning (Huber et al., 2016).

The experiment used the three-peg, three-disk version of the ToH puzzle. The 3D version was a traditional, timber incarnation of the ToH which consisted of natural wooden-looking pegs; and three wooden disks, each a different color and size (small, medium, and large). The 2D version of the ToH task was performed on a commercially available, iPad application (“Extra Tower of Hanoi” by Morard Dany).

**FIGURE 1:**
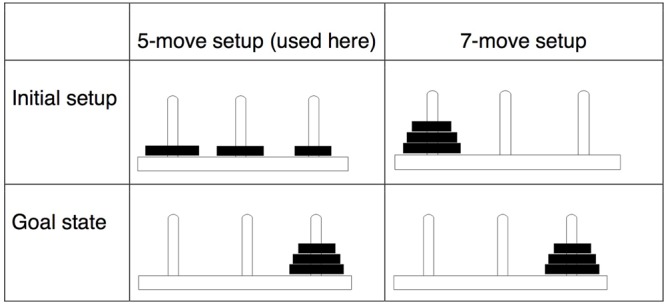
Initial state and goal state of the 5-move and 7-move Tower of Hanoi puzzle.

To solve the ToH puzzle the child must move all three disks to one specific peg, while abiding by three rules: (1) only one disk can be moved at a time, (2) a disk cannot be placed on a smaller disk, and (3) the disks can only be placed on one of the three pegs (i.e., they cannot be put on the ground or table). **Figure 1** shows the initial state and the target state for the pegs.

Each child attempted to solve the ToH puzzle four times, as described below in the Procedure section. During each of those four trials, the child had a ToH set (or iPad running a ToH app) in front of them, while another set (2D or 3D as appropriate) depicting the goal state, sat across the table (in front of the experimenter) for the child to reference any time during the task.

Consistent with the reference study, we used the “monkey family” variation of instructions, based on Klahr (1978). The experimenter told the child that the disks were a family of monkeys: a father monkey (large disk), a mother monkey (medium-sized disk) and a baby monkey (small disk). The monkeys were described as “tired” so the task was to move them to their sleeping tree—the peg furthest to the child’s right. It was explained that only one monkey could leave the tree at time, and a bigger monkey could not sit on a smaller monkey. The instructions were provided in Croatian, the language in which the experimenter and participants communicated. Participants were continuously recorded using an unobtrusively placed camera.

### Procedure

Our procedure was the same as that used by Huber et al. (2016), with the exception that, for all children, the initial state of the puzzle was set up with the first two moves pre-completed (see **Figure 1** above) such that it could be optimally solved in five moves (with each extra move indicating less optimal performance). This varied slightly from the reference study, in which children were assigned to either a 5-move or 7-move version of the puzzle depending on how they performed on a pretest probe trial. In the current study, we focused on the 5-move version because our participant pool included younger children who were very unlikely to succeed at the 7-move version even after extended practice.

We randomly assigned each child to one of three experimental conditions as follows:

• In the first condition, 3D-3D-3D-3D (or “No-transfer Condition”) we had the children attempt the task on the physical, 3D version of the puzzle on all four trials. This condition served as a baseline to demonstrate how children generally perform when no transfer of knowledge across modality is necessary (*n* = 17, age: *M* = 4.7, *SD* = 1.1).
• In the second condition, 3D-2D-2D-3D (or “Transfer Condition”), we had the children attempt the 3D trial initially, followed by two trials using the 2D version (i.e., with the ToH iPad app), and finally use the 3D version in the last trial. By comparing children in this condition to those in the 3D-3D-3D-3D condition, we probe the extent, if at all, practicing in the virtual modality affects performance afterwards in the 3D modality (*n* = 16, age: *M* = 4.9, *SD* = 1.2).
• The third condition, 2D-2D-2D-3D (or “No Pre-exposure Condition”) is similar to the 3D-2D-2D-3D condition, except that children were never exposed during the study to the 3D version until the final trial. This condition is included to ascertain if pre-exposure to the 3D version is necessary for children to effectively learn from the 2D version and/or apply learning in the 2D version back to the 3D version (*n* = 16, age: *M* = 4.8, *SD* = 1.2).

The protocol was approved by ethics board at University of Rijeka and undertaken conforming to the regulations. All children who participated did so with the written informed consent of at least one parent or guardian.

### Coding and Analyses

From the video and screen recordings we coded all disk moves in each of the four trials for each child. For each trial, for each child, we calculated the time to complete the task and the number of moves used to complete the test. In trials where the children solved the puzzle within the given 5-min period, we recorded the time taken to complete the puzzle; and if the child did not solve the puzzle, we recorded 5 min as completion time. A move was defined as a child lifting a disk from a peg and placing it back on the same peg, or on to another. When the child violated any of the three rules (outlined in the Materials section), the experimenter informed the child of the rule break. In that case, we counted both the rule breaking move and the subsequent correcting move as separate complete moves.

We examined two dependent variables, “Total Moves” and “Time per Move.” Time per Move was computed by dividing the time by the number of moves. Total Moves was the number of moves the child made to complete the puzzle (or within the 5 min if they did not complete the task).

To assess coder reliability a second observer coded for Total Moves with randomly selected subset of participants (*n* = 33 trials). Krippendorff’s alpha for interval data was computed at 0.988 verifying a high level of agreement across observers. Fewer than 15% of the individual scores for any trial differed across the observers and when there were differences, there were no differences in the ranked order of the scores across the four trials.

## Results

### Total Moves Analysis

**FIGURE 2:**
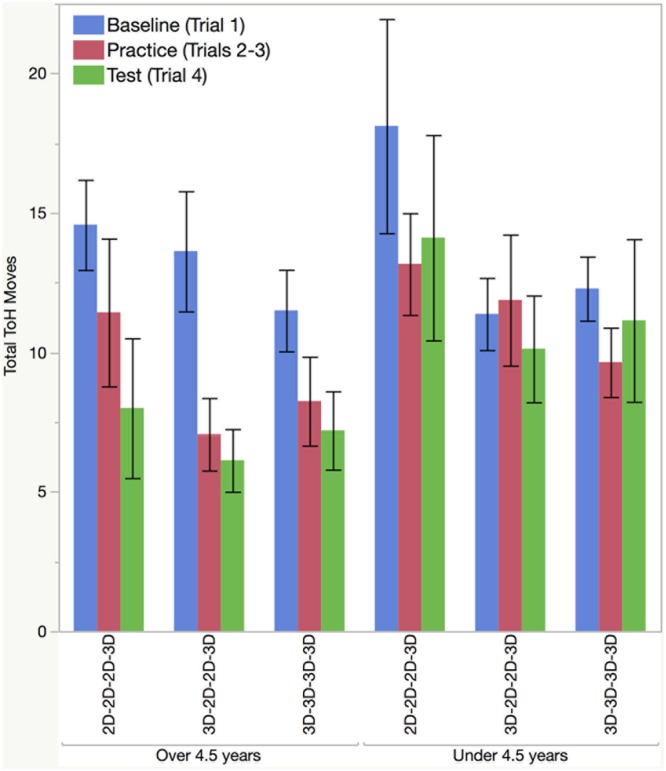
Number of moves taken to solve task by condition, trial, and age group. Analyses were performed on Trials 1 and 4. Practice trials bars are the mean of Trials 2 and 3. Error bars reflect standard errors.

**Figure 2** shows the Total Moves data. Data was analyzed using a full factorial repeated measures regression on Total Moves with condition and age (as a continuous variable) as between subjects predictors and trial number (1 vs. 4) as a within subject predictor. There were no significant main effects of condition, age, or trial. However, the analysis did reveal a significant age by trial number interaction, *F*(1,43) = 6.75, *p* = 0.01, η^2^ = 0.14. Further analyses demonstrated that the interaction was driven by the fact that older children improved from trial 1 to trial 4, but younger children did not improve. We confirmed this by examining the older and younger children separately with a matched pairs *t*-test, dividing the groups with a median split on age, resulting in relatively equal sized groups (*n* = 25, *n* = 24). Older children (*M* age: 5.6 years; *SD*: 0.66; Range: 4.58–6.5; 44% male), improved significantly from trial 1 to 4, *t*(24) = -4.01, *p* < 0.001, Cohen’s *d* = 0.80, whereas younger children (*M* age: 3.79 years; *SD*: 0.46; Range: 3.08–4.42; 46% male) did not improve, *t*(23) = 0.96, *p* = 0.35.

Huber et al. (2016) found that Total Moves decreased from the 1st to 4th trial, regardless of whether the practice trials were in 2D or and 3D modality. The results from the older group in the current study replicates this finding. However, in the current analysis the age range for the older group (4.58 to 6.50 years) differed somewhat from the reference study (4.05 to 6.50 years). For a more precise comparison to the reference study, we applied an ANOVA on Total Moves for all children older than 4 years of age. This ANOVA used condition as a between subjects predictor and trial as a repeated-measure. Consistent with Huber et al. (2016) there was a significant effect of trial, *F*(1,33) = 16.70, *p* < 0.001, Cohen’s *d* = 0.68, but no effect of condition (*F* = 1.35), nor a trial by condition interaction (*F* = 0.62).

### Time per Move Analysis

Data was analyzed using a full-factorial repeated measures regression on Time per Move with condition and age as between subjects predictors and trial number (1 vs. 4) as a within subject predictor. The results of this analysis revealed a main effect of age, *F*(1,43) = 17.8, *p* < 0.001, with moves being made, on average, 0.99 s faster with each year of age. Additionally, it revealed a trial by condition interaction, *F*(1,43) = 3.43, *p* = 0.04. This interaction reflects that children in the 3D-3D-3D-3D condition improved their move speed by 4.36 s from trial 1 to trial 4, whereas the 3D-2D-2D-3D and 2D-2D-2D-3D conditions improved only by 0.93 and 0.83 s, respectively. Given that the 3D-3D-3D-3D group had the most practice moving disks in a single modality, this effect is not surprising. It is also consistent with the original finding that children in the 2D-2D-2D-3D condition did not become significantly faster from baseline to test. There were no other significant main effects or interactions resulting from this analysis.

Because the reference study did report a significant effect of trial, with children making moves more quickly by trial 4, an additional analysis was conducted with children aged over 4 years (consistent with the reference study). An ANOVA with condition as a between subjects factor and trial as a repeated measure revealed a significant effect of trial for this group of 4- through 6-year-olds, *F*(1,46) = 10.29, *p* = 0.002, Cohen’s *d* = 0.74.

## Discussion

The main contribution of this work is the verification that children over 4 years of age can learn to solve a problem using a touchscreen app and transfer this learning to solve an isometric problem in the physical world. This finding, originally reported by Huber et al. (2016) with Australian children, is replicated here with a sample of preschool children in Croatia.

Huber et al. (2016) studied children ages 4 to 6 years of age. Total Moves and Time per Move significantly decreased from the initial baseline trial to the final test trial. This was the case, regardless of whether the children practiced the task in the 2D or 3D modality. The current study confirms these findings.

The similarity in results across the two studies underscore the validity of a number of points made in the reference study. In particular, the findings that children smoothly transferred the problem-solving skill that they practiced in 2D to apply to the 3D model illustrates the limits of ‘screen time’ as a construct. ‘Screen time’ does not distinguish activities that involve active engagement from those that involve only passive viewing. While children may have problems learning problem-solving strategies from certain screen-based activities, the current and reference studies demonstrate that not all screen time has the same learning value.

Indeed, the current task appears to require cognitive engagement adequately complex to result in problem-solving learning (e.g., Bauer and Mandler, 1992). Consistent with these findings are those of Wang et al. (2017), which report that 5- to 6-year-old children, learned how to tell time from a touchscreen time-telling app and then apply what they had learned from the touchscreen to a toy clock. Both our tasks and theirs required children to focus on rules and thus contrast imitation tasks where greater attention may be given to the superficial differences around modality.

In the current study, the children under 4 years of age showed little ability to improve at the ToH problem regardless of the practice modality. That finding may result from using a task that is not suitable for children of that age. For further research with the younger age group, a suitable option may be to use a different but common implementation of the ToH task, i.e., begin with the 2-disk version, then the 3-disk version, and increase the number of disks by one until the child cannot complete task. The performance variable would be scored as the greatest number of disks with which each child successfully completed the puzzle.

While the current experiment verifies the finding of the reference study, it is notable that in both studies children received instructions about how to complete the task from a live experimenter. They were not simply given a touch screen device and left to learn the task alone. Zack and Barr (2016) demonstrated the impact of adult scaffolding when young children use a touch screen device, with 15-month-old infants more likely to transfer learning between a touch screen device and a physical object when they had high levels of scaffolding. Future research could build on the current study and manipulate how the initial instructions are given—via a touchscreen app or from live experimenter. This could address whether the social interaction involved in the procedure impacts the children’s learning.

Finally, the replication of results despite the study originally being undertaken in Australia, and this time in Croatia strengthens the validity of the findings. Furthermore, transferring the problem-solving skills to complete the ToH task has now been demonstrated by both English speaking and Croatian speaking children.

## Conclusion

This study replicates the findings of the Huber et al. (2016) study and showed that children 4 years and older can transfer learning from 2D to 3D, even without exposure to 3D prior to the 2D exposure. We found that children under 4 years do not appear to improve their ability to solve the ToH problem with either the touch screen or the physical model.

## Author Contributions

Conceived and designed the experiment: JT and JK. Performed the experiment: AD. Analyzed the data: JT and JK. Wrote the paper: JT, JK, and AD.

## Conflict of Interest Statement

The authors declare that the research was conducted in the absence of any commercial or financial relationships that could be construed as a potential conflict of interest.
